# Terahertz Spatiotemporal Wave Synthesis in Random Systems

**DOI:** 10.1021/acsphotonics.3c01671

**Published:** 2024-01-09

**Authors:** Vittorio Cecconi, Vivek Kumar, Jacopo Bertolotti, Luke Peters, Antonio Cutrona, Luana Olivieri, Alessia Pasquazi, Juan Sebastian Totero Gongora, Marco Peccianti

**Affiliations:** † Emergent Photonics Research Centre, Department of Physics, School of Science, Loughborough University, Loughborough LE11 3TU, U.K.; ‡ Emergent Photonics Lab (EPic), Department of Physics and Astronomy, University of Sussex, Brighton BN1 9QH, U.K.; § Department of Physics and Astronomy, University of Exeter, Exeter, Devon EX4 4QL, U.K.

**Keywords:** terahertz, scattering, wavefront shaping, superfocusing, random media, genetic algorithm

## Abstract

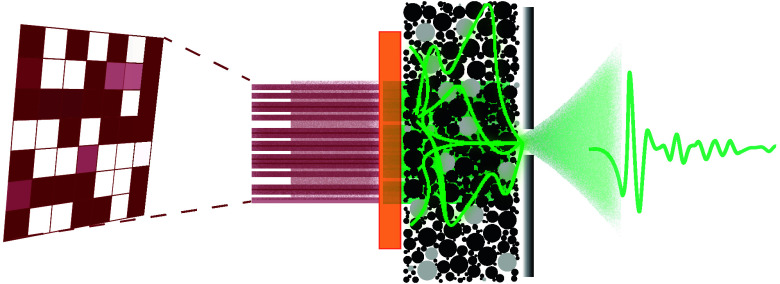

Complex media have emerged as a powerful and robust framework to control light–matter interactions designed for task-specific optical functionalities. Studies on wavefront shaping through disordered systems have demonstrated optical wave manipulation capabilities beyond conventional optics, including aberration-free and subwavelength focusing. However, achieving arbitrary and simultaneous control over the spatial and temporal features of light remains challenging. In particular, no practical solution exists for field-level arbitrary spatiotemporal control of wave packets. A new paradigm shift has emerged in the terahertz frequency domain, offering methods for absolute time-domain measurements of the scattered electric field, enabling direct field-based wave synthesis. In this work, we report the experimental demonstration of field-level control of single-cycle terahertz pulses on arbitrary spatial points through complex disordered media.

The physics of wave propagation in multiple scattering environments has been of interest in various domains ranging from solid-state physics to optics and seismology, whether in quantum or classical regimes.^1−4^ The study of the statistical moments of the transmitted wave amplitude resulted in the definition of many physical parameters of scattered fields that enabled the investigations of waves within complex systems, e.g., the mean free path, the diffusion constant, or the dimensionless conductance—also known as the Thouless factor. Empirical manifestations of multiple scattering in diffusive regimes, such as coherent backscattering, long-range correlation, and wave localization, have also been observed for different kinds of waves (e.g., acoustic, plasma).^5−9^ Unlike other domains, like acoustics, where the number of spatially independent modulation points is relatively small, at optical frequencies, spatial light modulators (SLMs) offer millions of degrees of freedom to control the propagation of coherent light. This has been a pivotal element in studying transformations based on complex optical systems.

Multiple scattering in the Mie regime, when wavelength and scattering elements lie at similar dimensional scales, is generally perceived as structural disorder associated with a loss of net transmitted information. For instance, when coherent light propagates in an inhomogeneous medium, its wavefront is disorganized into complex patterns with no trivial connection to the incident input field, hindering, for example, imaging capabilities. It is worth noting, however, that this process does not generally alter the phase coherence of the transmitted light, and scattered waves arriving from “different paths” through the sample interfere with one another. The random occurrence of this phenomenon in space is associated with the emergence of laser speckles with coherent light. This phenomenon is deterministically associated with a fixed scattered morphology (although complex).^10^ We can argue then that scattering media behave as a complex combinatory element that provides access to a large number of distinct space-time transformations. Specific spatial input illuminations can then couple specific modes of the scattering medium, yielding the coherent superposition of a defined set of those transformations. For a sufficiently complex medium, seeking an apt approximation of a desired function in this set becomes possible.

Pioneering works in this direction were laid out by A. Mosk and co-workers,^11^ in particular, within the framework of imaging mediated by disordered systems.^12,13^ Adaptive and computational imaging processes based on iterative methodologies have been successfully applied to light diagnostics.^14−16^ The access to specific scattering-driven functionality has been realized using spatial-light modulation of the illuminating field. Different approaches have been proposed to retrieve the optimal illumination pattern associated with a target field distribution at the output of the scattering system, including feedback-driven optimization,^17−19^ optical phase conjugation,^20,21^ and measurement of the medium transfer matrix.^22,23^

To provide a gateway to field synthesis at optical frequencies, the combination of interferometric setups and tunable monochromatic sources has been employed to infer the complex spectral-phase transformation induced by the scattering media,^11^ as it potentially enables arbitrary manipulation of amplitude and phase of fields for a known input excitation. Parallel research tackled the nontrivial problem of disorder-induced temporal broadening, an inherent challenge connected to the propagation of ultrashort broadband pulses in disordered media. Remarkably, recent results have suggested that the intrinsic spatiotemporal coupling induced by scattering media enables the control of the temporal properties of the scattered field (i.e., the full-wave synthesis through the scattering system^24,25^) through spatial-only wave modulation. However, it is essential to note that the simultaneous control of multiple frequencies remains challenging in the optical domain because of fundamental and practical implementation limits.

A suitable platform can be sought in the THz domain, where a field-sensitive detection scheme known as THz time-domain spectroscopy (TDS) is well-established and permits the time-resolved detection of electric fields from single-cycle THz pulses.^26^ Field detection has been successfully deployed to probe the broadband properties of scattering samples and retrieve detailed knowledge of the complex scattered spectral fields.^27−29^ Although time-domain studies of scattered THz waves have been tackled in the art,^30^ the interest in spatiotemporal control has steadily increased. Recently, we theoretically established a novel methodology to achieve space-time control of scattered broadband THz pulses.^31,32^ This paper can be considered the seminal experimental proof of those works: in this study, we demonstrate the full-field control and optimization of the transmitted THz pulses utilizing a genetic algorithm, i.e., a field-based wave synthesis on arbitrary points.

Notably, refs (31 and 32) depict scattering as a dimensionless optical combinatory system for numerical convenience^33^ whereas our experiments further prove that near-field coupling effectively provides access to significant medium complexity and a sufficient number of orthogonal modes in specific scatterer-builds, a particularly challenging aspect to predict from abstract scattering models.

## Methods

Our methodology takes advantage of the Time-domain Nonlinear Ghost Imaging (NGI) approach,^34−37^ a framework we developed to sample the spatiotemporal response of an object at a deeply subwavelength scale. NGI is based on the nonlinear conversion of spatially patterned ultrafast optical pulses, which is a crucial feature in this work. Notably, by combining the nonlinear generation THz patterns from randomly structured optical beams with an evolutionary optimization feedback scheme, we predicted control over the scattering field and managed to focus and manipulate the broadband THz radiation throughout a random system.^31^

In a general description, we can assume a scattering medium defined by a space-frequency transfer operator *TM*(*x,x*′,*y,y*′,ω) (as per refs (38−40)). For the sake of simplicity, we retain a simple scalar description (which, however, remains valid in a full vectorial formulation^41^) and denote the THz input field distribution *E*^–^(*x*′,*y*′,ω) and the field transmitted through the scatterer *E*^+^(*x*,*y*,ω) as follows:
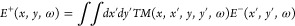
1where we define (*x*′, *y*′) and (*x*, *y*) as the coordinates at the input and output planes, respectively. Within the framework of optical wavefront control, our aim is to identify the optimal THz incident field distribution *E*_*optimal*_^–^(*x*′,*y*′,*t*) which produces a desired THz transmitted field *E*_*target*_^+^(*x*,*y*,*t*) at the output facet of the scatterer.^31^ While operating in the terahertz band creates agile access to the instantaneous electric field, the ability to control the incident electric field distribution is hindered by the limited availability of SLM devices at those frequencies.

As a critical difference from the optical domain, the THz wavelength is quite long, which results in a very coarse diffraction limit. In optical embodiments, illuminating the surface of a scattering medium with a fraction of a millimeter-scale spot size guarantees a significantly complex optical combinatory process, i.e., the existence of several independent modes of the medium accessible to the impinging light. By comparison, at THz frequencies, this framework would translate to an exceedingly large (unrealistic) illumination setting.

The solution to both aspects, pursued via Time-domain Nonlinear Ghost Imaging, is to perform the nonlinear conversion of a structured optical beam in the near-field of a scattering medium.^34−36^ Any optical pattern generated through a standard SLM device can act as a direct source of THz patterns, which can be deeply subwavelength defined. Because the SLM shapes the optical pump, which is later transformed into a terahertz waveform using nonlinear conversion, this releases our control methodology from the traditional constraints of aperture-based wavefront shaping.^42,43^ In addition, near-field coupling allows access to a large spectrum of volume modes of the scattering medium that cannot be coupled via far-field illumination. This translates into the availability of a much larger set of independent transfer functions for a given wavelength-normalized scattering density.

By considering a quadratic process in a nonlinear crystal, the relation between the spatial distribution of the incident optical intensity and generated THz field is expressed as
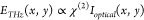
2enabling precise control over the THz field profile by simply shaping the incident optical pulse with a spatial spectrum limited to the much smaller optical wavelength. The optical-to-THz nonlinear conversion ensures the generation and control of single-cycle THz subwavelength patterns because they are bounded to the much finer optical diffraction limit.

**Figure 1:**
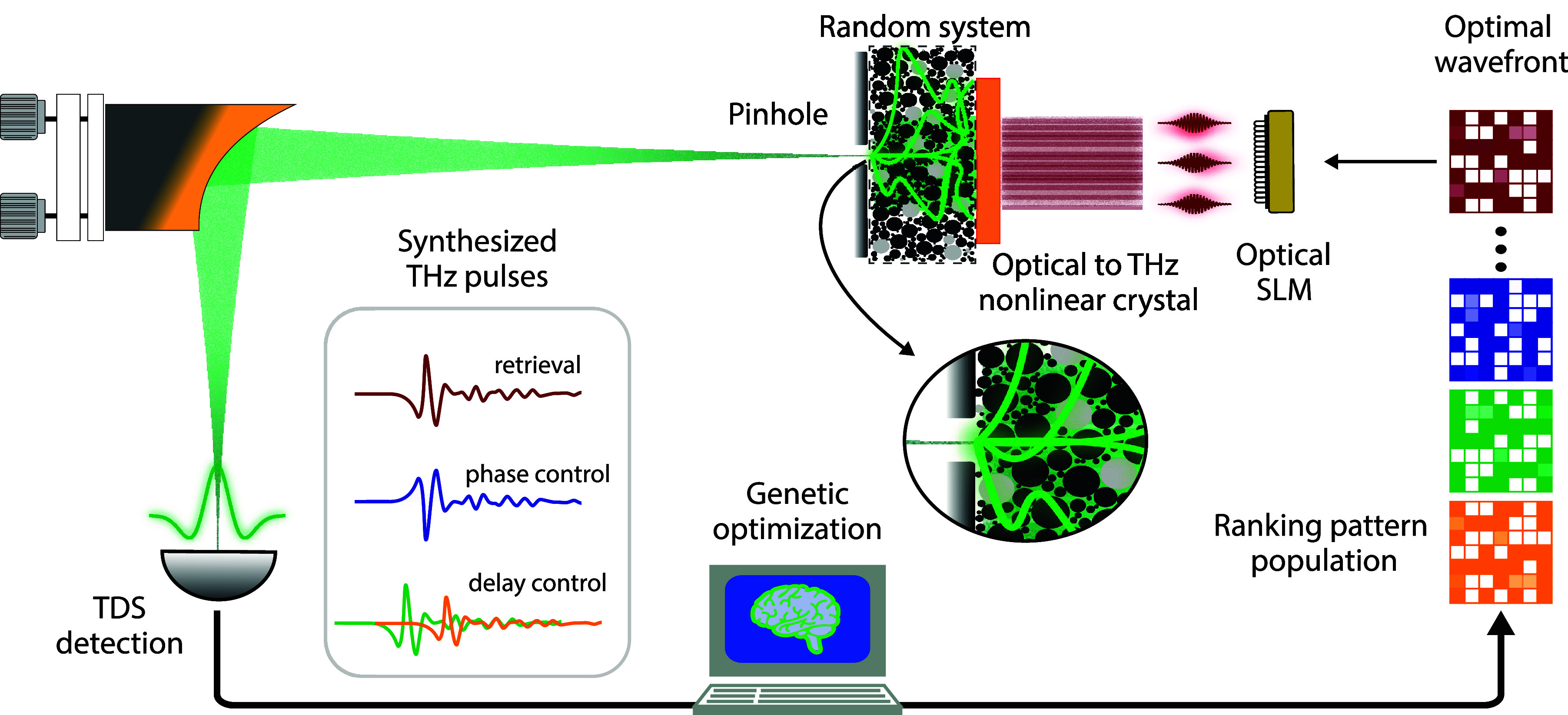
Conceptual overview of the ultrafast terahertz (THz) pulse synthesis. By using a standard time-domain spectroscopy (TDS) detection scheme, we collected the coherent transmitted field throughout the scattering medium. The methodology is based on a genetic algorithm optimization to achieve a desired linear transformation between the input and output fields, i.e., to synthesize the terahertz pulses throughout the random system.

The main components of our experimental setup are schematized in Figure 1. We employ ultrafast pulses with a duration of about 90 fs and energy of 1 mJ, centered at the wavelength λ = 800 nm generated by a Coherent Libra-HE Ti:Sa regenerative amplifier (modulated into a pattern of 32 × 32 pixels for an area of 6.4 mm × 6.4 mm). The pattern is converted into a distribution of THz sources via optical rectification in a nonlinear crystal (ZnTe) near-field coupled with a scattering medium.^44^ The experimental setup details and a description of the scattering media used in this study are available in the Supporting Information.

In this investigation, our proof-of-concept is the manipulation of the full-field properties of the transmitted THz pulse in a selected target location throughout a scattering system. Operatively, we isolate the scattered field from a specific output position via a pinhole placed in contact with the output facet of the scattering medium (see Figure 1), which is imaged onto our time-domain sensor (i.e., electro-optic sampling). To optimize the waveform of the transmitted pulse, we made use of a genetic algorithm. More specifically, its fundamental steps are as follows: (i) The creation of a population of amplitude patterns imaged on the THz crystal via a spatial light modulator. (ii) The measurement of the cost function of each pattern and the ranking of projected patterns. (iii) Implementing breeding, with the parent pattern being selected with a higher probability if it is more highly ranked, and random mutations of the offspring patterns with a mutation rate exponentially decreasing across different generations. (iv) The measurement of the cost function of the new offspring and its placement within the population to replace the less-performing parent patterns. (v) The process is repeated for a fixed number of iterations until a satisfactory solution is achieved. A distinctive feature of this approach is that all of the cost functions and their deviations are written in terms of time-domain field waveforms.

The optimization seeks to maximize a cost function which is calculated upon the time-varying electric field^31^ at the desired spatial point, i.e. *E*_0_(*t*) ≡ *E*(*x*_0_,*y*_0_,*t*). In Table 1, we define the cost functions used in the genetic algorithm in terms of statistical quantities defined as follows:
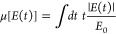

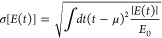
with . The quantities μ[*E*(*t*)] and σ[*E*(*t*)] correspond respectively to the temporal center and duration of the transmitted THz pulse waveform. For the sake of clarity, we stress that *E*(*t*) is the real time-varying field, so |*E*(*t*)| is not the pulse envelope but just the modulus of the electric field.

**Table 1:** Cost Functions

*Cost Function*	*Definition*	*Optimization Type*
A	*max*[*E*_0_(*t*)]/σ[*E*_0_(*t*)]	Spatiotemporal focusing
B	–*min*[*E*_0_(*t*)]	Phase inversion
C	–|μ[*E*_0_(*t*)] – *t*_0_|	Delay-shift

Interestingly, because of how those cost functions (CFs) are formulated, as compared to typical optical embodiments, an optimization upon the field, as in CF-A, also affects the absolute phase of the pulse.

## Results and Discussion

**Figure 2:**
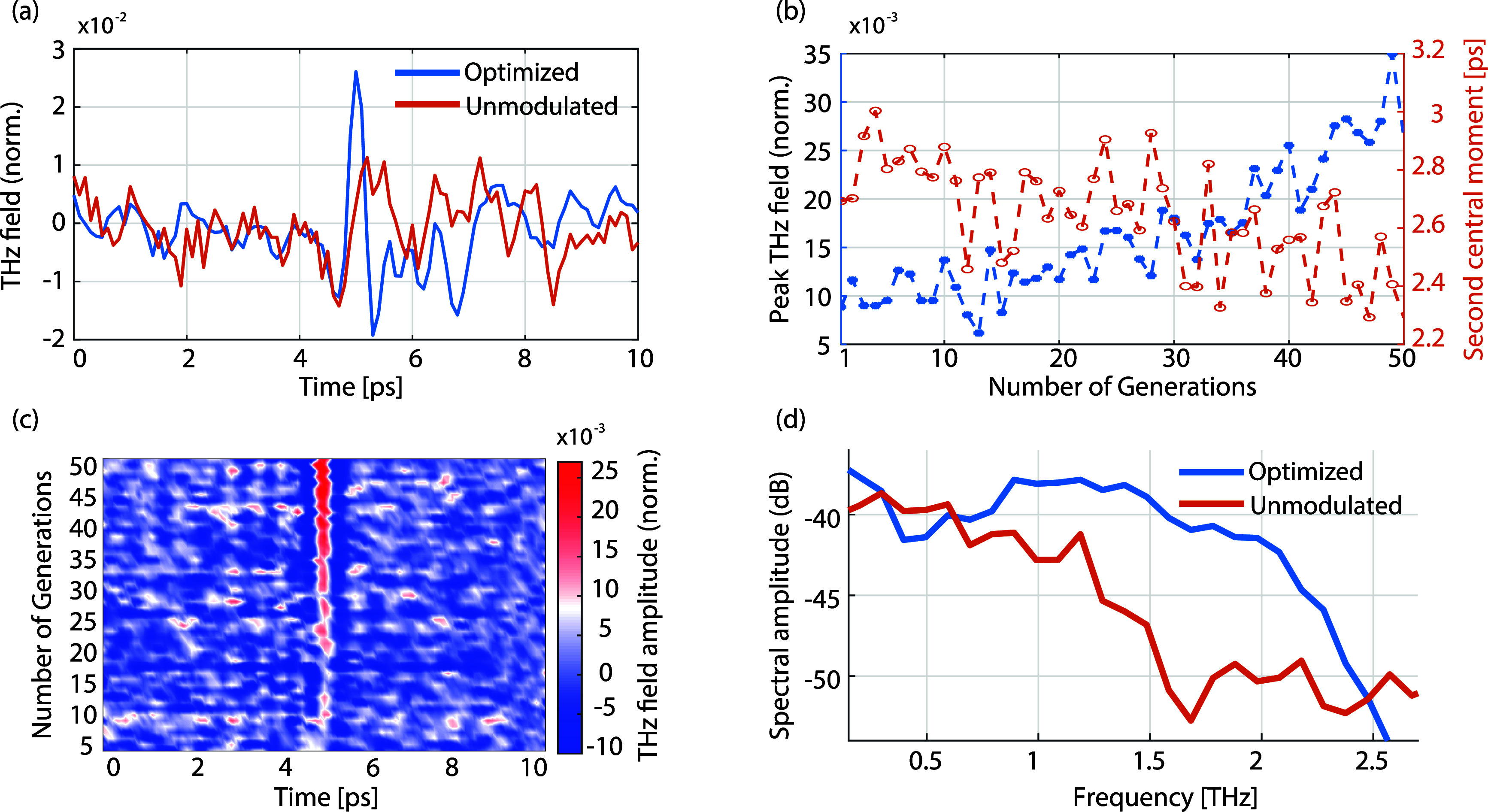
Spatiotemporal focusing of a terahertz pulse. (a) Optimized pulse (blue) and unoptimized field with unmodulated pattern (red). (b) The second central moment and peak field value optimization. (c) The set of time-domain spectroscopy time traces collected during optimization. (d) Spectra of the optimized and unoptimized pulses. All data normalized to the peak of the input pulse.

Figure 2 presents the spatiotemporal focus of an ultrafast single-cycle THz pulse obtained via optimization of the cost function A. As shown in the theoretical work in ref (31), the optimization has the effect of recovering a transform-limited pulse with a null carrier-envelope phase because of the higher instantaneous peak field and low pulse width (Figure 2(a)) expressed in this condition (i.e., a Ricker waveform). This is the case also in the optical domain when the peak temporal intensity is maximized through a nonlinear product.^45^Figure 2 depicts a comparison between the optimized pulse and the unmodulated pulse (i.e., with all the SLM pixels on) with normalization based on the peak of the input pulse (i.e., the pulse measured without the presence of the scattering medium).

The unmodulated illumination (approximately a flat Gaussian beam profile) produces a temporally broad random THz waveform. As evidenced in Figure 2(b), the second central moment of the field distribution reduces as the peak field increases. We stress here that the momentum is an assessment not of the pulse envelope (as typically done in optics) but of the absolute field distribution. Figure 2(c) displays the collection of time traces for TDS during the optimization. In essence, it compares a generally decorrelated temporal waveform with the optimized one. Quite importantly, although the optimization recovers transmitted bandwidth (Figure 2(d)), the time-bandwidth produced actually reduces (the second-order momentum of the unoptimized pulse is within the scale of the full.

It is worth noting that, as typical in this space-time scattering optimization, the scattered field exhibits a −40 dB transmission. This is expected as (i) in transmission setups the backscattering component (unrecoverable) tends to be dominant and (ii) by optimizing the peak field (and not just the average intensity) we automatically introduce a selection of the modes with synchronized group delay.

**Figure 3:**
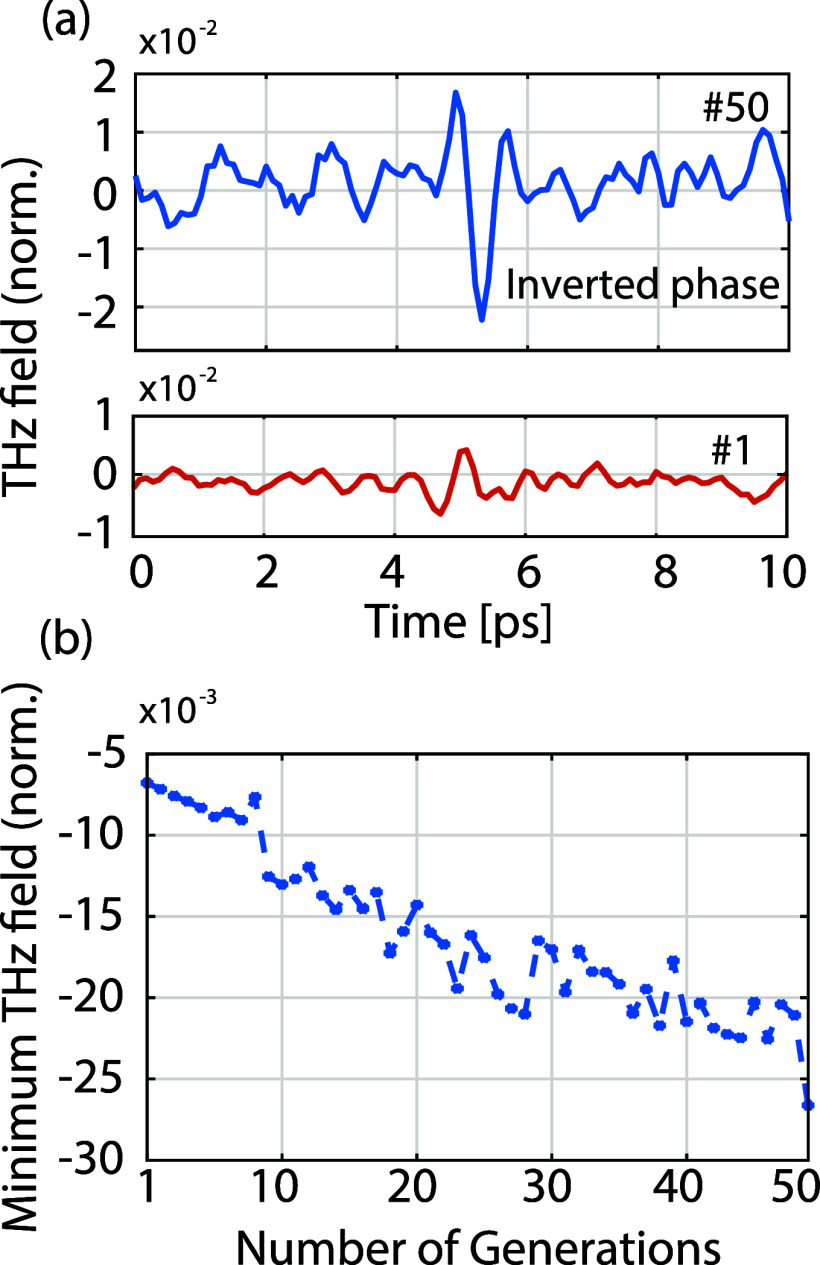
Phase inversion optimization. (a) Temporal profile of the output terahertz pulses at the first (red) and last (blue) iterations. (b) Minimum value of the field for the best performing pattern during optimization. All data normalized to the peak of the input pulse.

Moreover, as a proof-of-concept of our ability to control the phase of the transmitted pulse, we sought the best spatial pattern that minimizes the THz peak field through the cost function CF-B. This specific example allows us to demonstrate our control over the field sign. The corresponding temporal profiles are shown in Figure 3(a), where we can observe that the algorithm was able to flip the temporal trace of the pulse (in blue), compared to Figure 2(a). In Figure 3(b), we show the value of the cost function for the best-performing patterns across 50 generations.

Quite remarkably, the sensitivity of the detection to the absolute time delay enables us to show that the cost functions CF-A and CF-B lead toward a transform-limited version of the pulse centered at a slightly different time delay (CF-A and CF-B do not contain any direct reference to the temporal position of the pulse).

**Figure 4:**
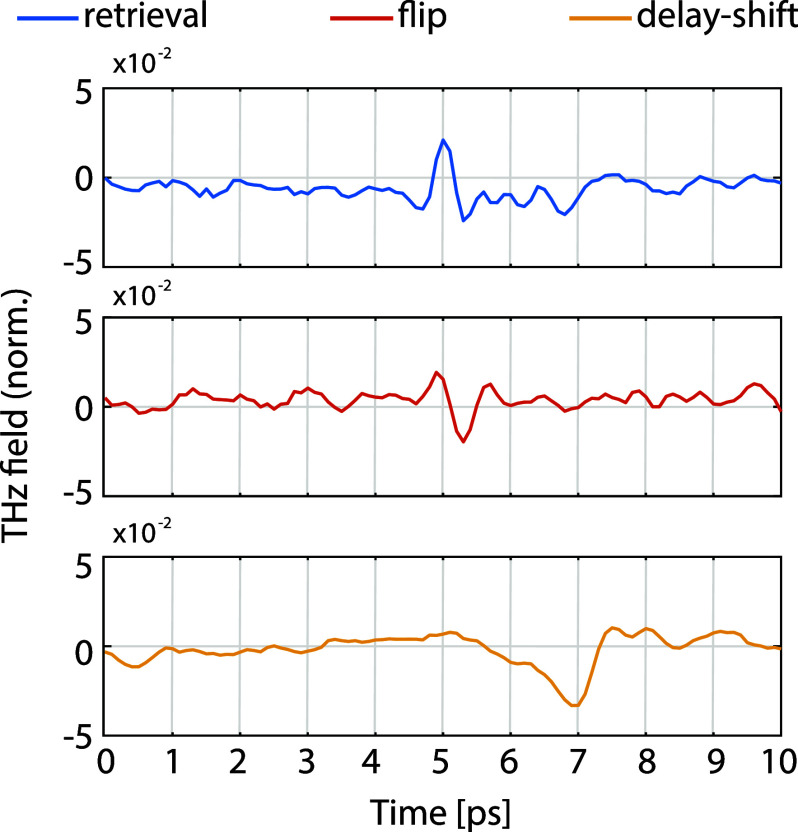
Field temporal shift. Comparison of the two optimized terahertz pulses and the time-shifted solution from CF-C. All data normalized to the peak of the input pulse.

By defining a CF as a function of μ[*E*(*t*)], we can explore our control over the absolute time delay of the pulse. Generally, if the medium is complex enough, it can expose available compounded transfer functions with a broad spectrum of potential group delays. For this scenario, we employed the optimization cost function CF-C, which seeks incident patterns yielding an output wavefront with the target temporal mean *t*_0_. Figure 4 shows that the pulse is successfully time-shifted relative to the previously optimized pulses. We stress that CF-C does not constrain any other pulse feature, so the temporal shift significantly changes the pulse profile. However, in the example of Figure 4, the shift is quite significant compared to the period of the optimized spatiotemporally focused pulse (blue plot). A low number of “modes” available with a large group delay is then expected. In other words, when dealing with significant delays, most modes exhibit nearly zero field transmission, limiting the available modes. Hence, the greater the desired delay, the more challenging it becomes to control a particular waveform.

This behavior is in good qualitative agreement with the experimental results of ref (46). Interestingly, in the framework of THz time-domain spectroscopy, these results suggest that scattering systems could be used to scan the THz pulse profile within a range of a few picoseconds without mechanical time-delay devices commonly used in ultrafast optical setups.

To our knowledge, this is the first work that showcases full field control in a complex system. The substantial advancement lies in the coherent measurement of the scattered electric field at a specific target point. While in the optical domain, it is possible to reconstruct the spectral phase of the transmission matrix using inverse interferometric reconstruction,^39^ this information alone does not enable the synthesis of a waveform without field-level knowledge of the source. The core finding is that field-level control is demonstrated through a scattering-driven combinatory process. We stress that while the scattering-based process can result in losses, this is common for many terahertz filter-based pulse-shaping solutions, and our demonstration shows access to an exceptionally large and reasonably continuous spectrum of accessible waveforms defined at the field level.

Also, unlike traditional spatiotemporal focusing at optical and infrared frequencies, our work leverages THz-TDS detection, providing a direct and coherent measurement of the transmitted electric field’s properties. This result holds profound implications, where time-resolved characterization techniques are highly sought after, including time-reversal control of optical waves.^47^

From recent studies on metamaterial analog computing,^48^ the proposed methodology has the potential to enable wave-based computational methodologies at THz frequencies.

In summary, our research presents a framework for the arbitrary spatiotemporal synthesis of THz waves through random media, enabling field-level control through a scattering-driven combinatory process.

## Supporting Information Available

Detailed schematic and description of the experimental setup (PDF)
