# Supplementary material for: Terahertz Spatiotemporal Wave Synthesis in Random Systems
Source: ACS Photonics. 2024 Jan 9;11(2):362–8. doi: 10.1021/acsphotonics.3c01671 (PMC10885209; doi:10.1021/acsphotonics.3c01671)
Supplement: Supplementary file 1 — ph3c01671_si_001.pdf [file ph3c01671_si_001.pdf]

Supplementary Information
Terahertz Spatiotemporal Wave Synthesis in Random Systems

VITTORIO CECCONI[1,2], VIVEK KUMAR[2], JACOPO BERTOLOTTI[3], LUKE PETERS[1,2], ANTONIO CUTRONA[1,2], LUANA OLIVIERI[1,2], ALESSIA PASQUAZI[1,2], JUAN SEBASTIAN TOTERO GONGORA[1,2] AND MARCO PECCIANTI[1,2]

[1]Emergent Photonics Research Centre, Department of Physics, School of Science, Loughborough University, LE11 3TU, U.K.

[2]Emergent Photonics Lab (EPic), Department of Physics and Astronomy, University of Sussex, BN1 9QH, U.K.

[3]Department of Physics and Astronomy, University of Exeter, Exeter, Devon EX4 4QL, U.K.

*Corresponding author: m.peccianti@lboro.ac.uk

KEYWORDS terahertz, scattering, wavefront shaping, superfocusing, random media, genetic algorithm.

1. Experimental Setup

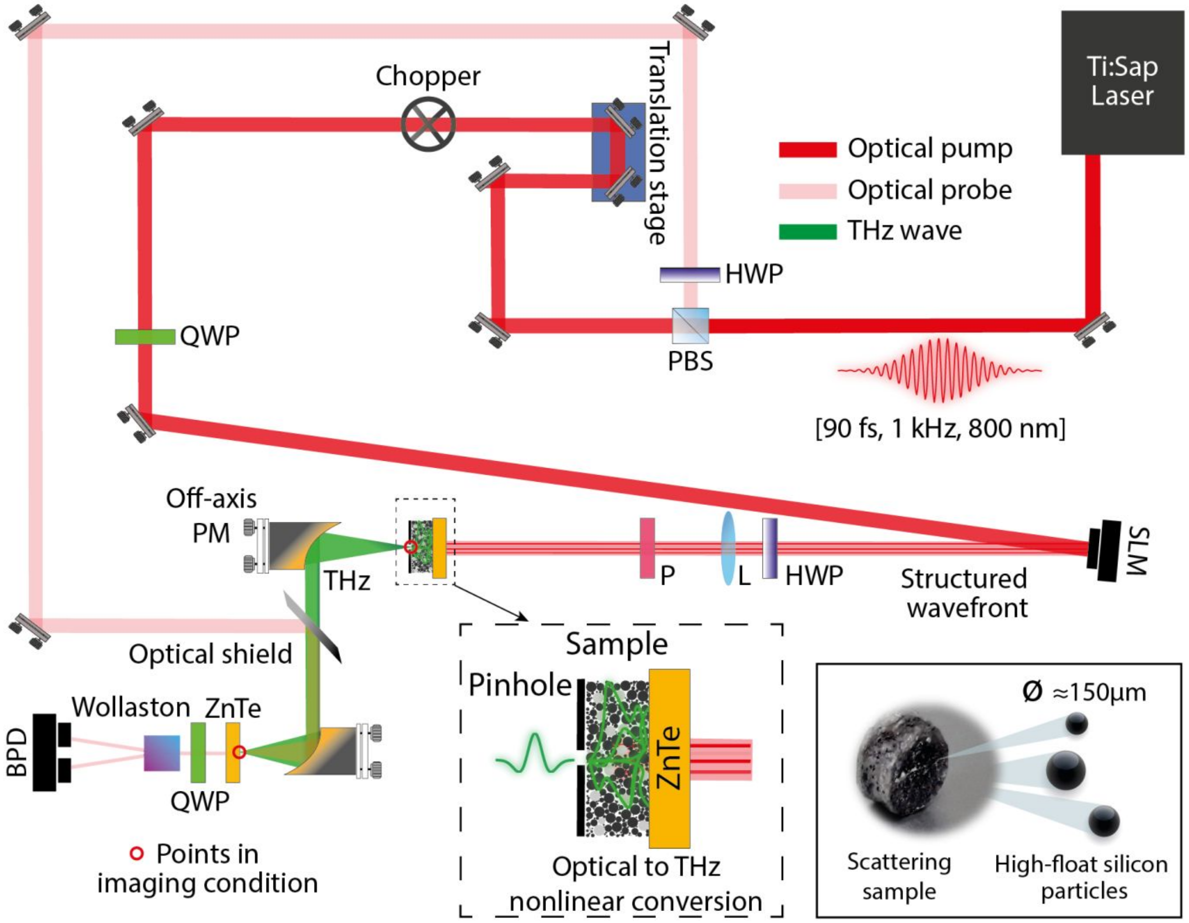

Figure S1. Schematic of the apparatus: Polarized beam-Splitter (PBS), Half Wave-Plate (HWP), Quarter Wave-Plate (QWP), Zinc Telluride (ZnTe), off-axis Parabolic Mirror (PM), Lens (L), Polarizer (P), Spatial Light Modulator (SLM); the optical shield was a High-float Silicon Wafer. The right inset shows a picture of the THz scattering sample.

In Fig. S1, we show the scheme of the experimental setup. A pulsed laser with a central wavelength of $800\ nm$ (Coherent Libra, $1\ kHz$ repetition rate) is reflected off an LCMOS-SLM (Hamamatsu X10468 Series). The SLM screen

is imaged on the surface of a zinc telluride (ZnTe) crystal, and the THz-shaped pattern is generated via optical rectification.

The patterns used in the experiment had a resolution of 32x32 pixels (for a physical modulated area of 6.4 mm x 6.4 mm). In this work, the laser energy was 1.0 mJ. The shaped THz wavefront is then projected on the scattering medium placed in contact with the nonlinear crystal to achieve near-field conditions. We placed a pinhole at the output of the scattering medium and the transmitted THz field is imaged with a 2f-2f imaging condition and coherently detected with a standard electro-optic detection (i.e., via a second ZnTe). The pinhole was made of aluminium foil with a $\sim 200\ \mu m$ aperture at the centre directly in contact with the scattering media output facet.
For both detection and generation, 1 mm ZnTe crystals with (110) orientations were used; the crystals were oriented for the p-polarization configuration. The static scattering medium (inset in Fig. S1) consists of (high-resistivity) float-zone silicon particles of around $150-300\mu\text{m}$ diameter embedded in a paraffin matrix with a Silicon mass fraction of 8% [1,2].
The time constant of the LOCK-IN amplifier was set to 300 ms, for an overall acquisition time of approximately 900 ms (i.e., considering the settling time). It took about 1.5 minutes to scan a complete field waveform. The overall optimization process took about 6.5 hours. Notably, the TDS is the most time-consuming action because of the specific setup at hand. However, sophisticated single-shot TDS implementation (beyond the scope of this paper) exists and would reduce the total process time by several orders of magnitude [3,4].

References

1. S. Gentilini, M. Missori, N. Ghofraniha, and C. Conti, "Terahertz Radiation Transport in Photonic Glasses," Annalen der Physik **532**(8), 2000005 (2020).
2. M. M. Qureshi, J. Brake, H.-J. Jeon, H. Ruan, Y. Liu, A. M. Safi, T. J. Eom, C. Yang, and E. Chung, "In vivo study of optical speckle decorrelation time across depths in the mouse brain," Biomed. Opt. Express, BOE **8**(11), 4855–4864 (2017).
3. E. Roussel, C. Szwaj, C. Evain, B. Steffen, C. Gerth, B. Jalali, and S. Bielawski, "Phase Diversity Electro-optic Sampling: A new approach to single-shot terahertz waveform recording," Light Sci Appl **11**(1), 14 (2022).
4. G. T. Noe, I. Katayama, F. Katsutani, J. J. Allred, J. A. Horowitz, D. M. Sullivan, Q. Zhang, F. Sekiguchi, G. L. Woods, M. C. Hoffmann, H. Nojiri, J. Takeda, and J. Kono, "Single-shot terahertz time-domain spectroscopy in pulsed high magnetic fields," Optics Express **24**(26), 30328 (2016).
